# Transport characteristics of a silicene nanoribbon on Ag(110)

**DOI:** 10.3762/bjnano.8.170

**Published:** 2017-08-16

**Authors:** Ryoichi Hiraoka, Chun-Liang Lin, Kotaro Nakamura, Ryo Nagao, Maki Kawai, Ryuichi Arafune, Noriaki Takagi

**Affiliations:** 1 Department of Advanced Materials Science, The University of Tokyo, 5-1-5 Kashiwanoha, Kashiwa, Chiba 277-8561, Japan; 2 International Center for Materials Nanoarchitectonics (WPI-MANA), National Institute for Materials Science, 1-1 Namiki, Tsukuba, Ibaraki 304-0044, Japan

**Keywords:** nanojunction, nanoribbon, scanning tunnelling microscopy, silicene, transport

## Abstract

We present the transport characteristics of individual silicene nanoribbons (SiNRs) grown on Ag(110). By lifting up a single SiNR with a low-temperature scanning tunneling microscope tip, a nanojunction consisting of tip, SiNR and Ag is fabricated. In the differential conductance spectra of the nanojunctions fabricated by this methodology, a peak appears at the Fermi level which is not observed in the spectra measured either for the SiNRs before being lifted up or the clean Ag substrate. We discuss the origin of the peak as it relates to the SiNR.

## Introduction

The electronic transport characteristics of nanomaterials from a single molecule, nanowires, nanotubes, and nanoribbons to two-dimensional (2D) atomic sheets have garnered much attention from fundamental and application points of view [1–7]. Silicene, a single-atom-thick honeycomb layer consisting of Si atoms, is one of such promising materials [8–13]. Freestanding silicene hosts the Dirac electronic system and behaves as a 2D topological insulator (TI) as a result of the sizable spin–orbit coupling of Si [14–16].

Silicene grown on solid substrates has been studied intensively. Various superstructures such as (4×4), (2√3×2√3)R30° and (√13×√13) R13.9° are formed on Ag(111) [17–22]. These structures are composed of buckled honeycomb configurations. However, they do not host Dirac fermions and do not exhibit the 2D TI features because of the interfacial coupling between the silicene layer and the substrate, as demonstrated for the (4×4) structure [23]. The key factor for realizing the 2D-TI silicene is to reduce the interfacial coupling. Recently, Tao et al. [24] successfully fabricated a silicene field effect transistor by peeling off the (2√3×2√3)R30° silicene from the Ag substrate and demonstrated the current–voltage characteristics supporting the survival of Dirac fermions. This study indicates the importance of reducing the interfacial coupling.

Not only a 2D sheet but also a 1D ribbon of silicene can be formed. Le Lay and collaborators have reported the formation of silicene nanoribbons (denoted as SiNRs hereafter) on Ag(110) [25–26]. The SiNR takes on the structure of a 1D honeycomb of ≈1.5 nm width with the zigzag edges. Very recently, pentagonal chain models were proposed for SiNR on Ag(110) [27–28]. In this model, Si atoms constitute a five-membered ring to form a 1D chain. Density functional theory (DFT) calculations have demonstrated that freestanding honeycomb SiNR preserves the electronic states localized at the edges near the Fermi level similar to the graphene nanoribbon with zigzag edges [29–33]. Although the electronic structure of SiNR has been studied experimentally [34–36], the existence of edge states remains an open question. The interfacial coupling between the SiNR and the substrate might modify the intrinsic electronic properties of SiNR as described above for the (4×4) silicene on Ag(111). Thus, it is required to decouple SiNR from the substrate and evaluate the intrinsic properties.

Here we report the transport characteristics of SiNR on Ag(110). To isolate SiNR from the Ag substrate, we lift up an individual SiNR with the tip of a low-temperature scanning tunneling microscope (STM) and fabricate a nanojunction in which the lifted SiNR bridges the gap between the STM tip and the substrate. This method enables us to isolate the SiNR from the substrate electronic system and elucidate the intrinsic properties. We measure the differential conductance (d*I*/d*V*) spectra of the nanojunctions and find a sharp peak structure at the Fermi level.

## Results and Discussion

**Figure 1:**
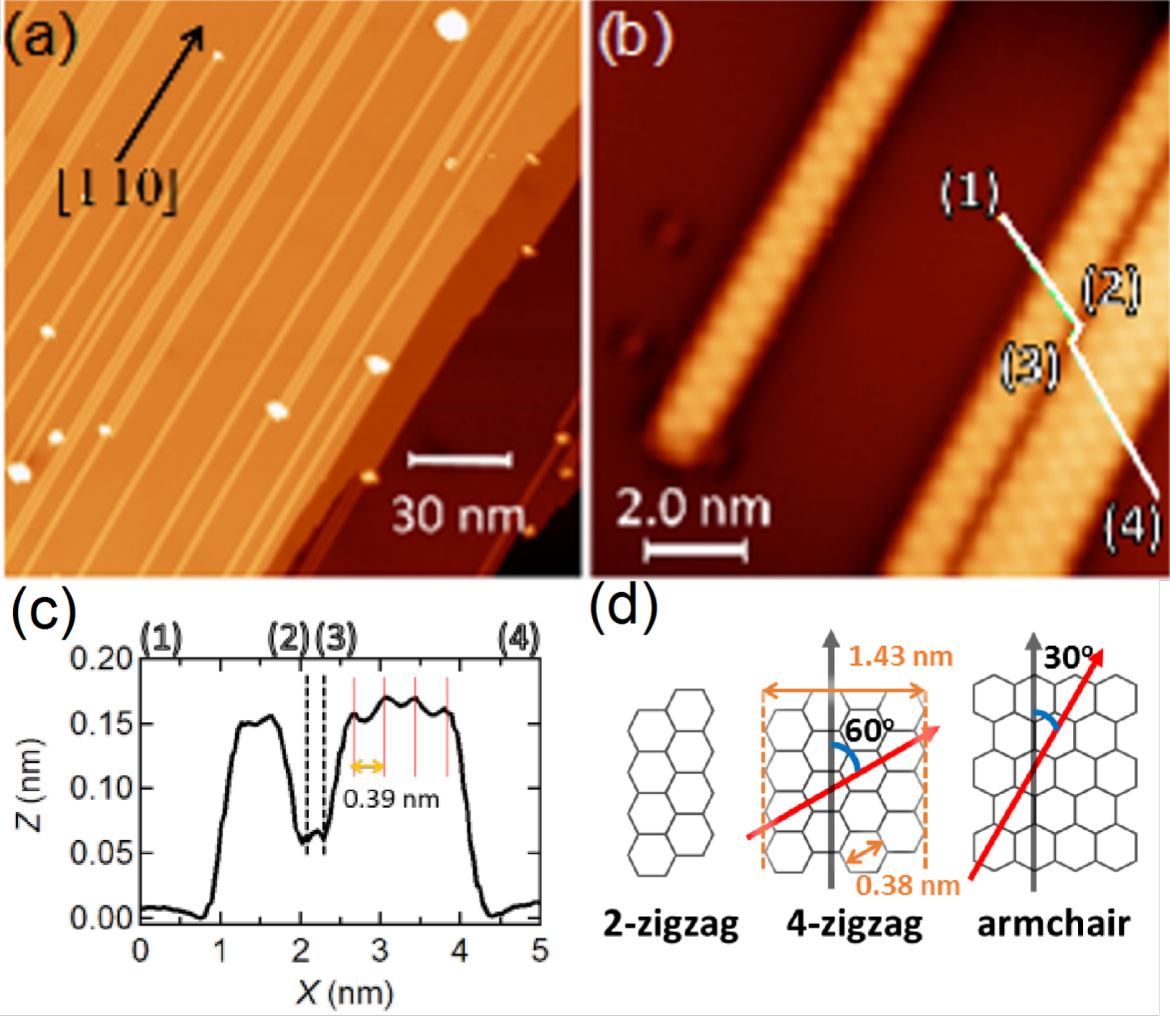
STM images of SiNRs on Ag(110) with (a) *V*_S_ = −500 mV, *I*_t_ = 10 pA and (b) *V*_S_ = −100 mV, *I*_t_ = 30 pA. (c) Cross-sectional height profile along the (1)–(4) line shown in (b). (d) Schematic structural models of SiNR structures. The black arrows dictate the longitudinal direction and the red arrows represent the row of the honeycomb units across the ribbons.

Figure 1a shows a topographic STM image of the Ag(110) surface after the deposition of Si atoms. The lines extend along the 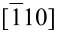 direction. Two types of lines are observed as shown in Figure 1b; one has a 1.6 nm width and the other a 0.8 nm width. The former and latter are composed of four and two bright spots across the longitudinal direction, respectively. The cross-sectional height profile in Figure 1c shows that the distance between the spots is 0.39 nm, which is nearly identical to the size of the honeycomb unit of Si. These features indicate that these lines are SiNRs with a zigzag edge structure, as demonstrated by the structural models in Figure 1d. The present results are nicely matched with those reported in the previous STM works [25–26].

**Figure 2:**
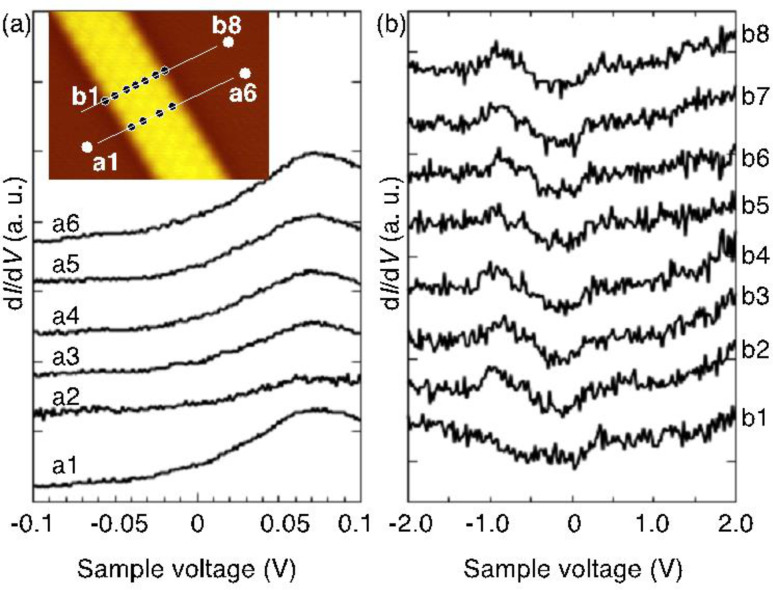
d*I*/d*V* spectra acquired in (a) narrow (from −0.1 to 0.1 V) and (b) wide (from −2 to 2 V) voltage ranges. The inset of (a) is an STM image showing the tip locations where the spectra are measured. The modulation voltage of 8 mV at 366.6 Hz is added to the sample voltage. Each spectrum is shifted vertically.

We measured d*I*/d*V* spectra as a function of the STM tip location. Figure 2a,b shows the spectra measured in the narrow and wide voltage ranges. One sees that these spectra are very similar to each other and do not depend on the tip location. The spectra taken for the SiNRs are almost the same as those for the Ag substrate. The spectra taken at the edges are essentially identical to those spectra taken inside the SiNR and do not show a spectral signature relevant to the edge. Similar to silicene on Ag(111), the interaction of SiNRs with the substrate may hamper the emergence of intrinsic electronic features of freestanding SiNR.

**Figure 3:**
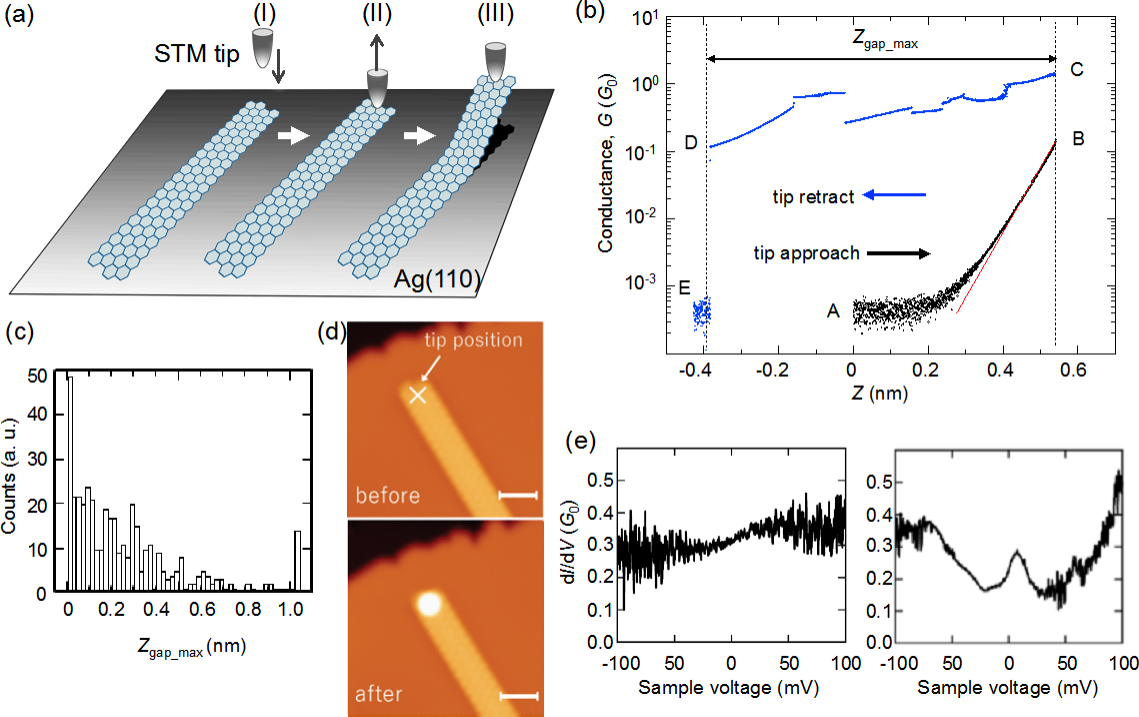
(a) Schematic illustration on fabrication of an SiNR nanojunction with an STM tip. (b) Conductance trace measured as a function of tip vertical position (*Z*). *G*_0_ is the conductance quantum (7.75 × 10^−5^ S). The feedback is turned off at *V*_S_ = 100 mV and *I*_t_ = 20 pA and the conductance is measured at *V*_S_ = 100 mV. The conductance measured during the tip approach and retraction procedures is plotted with black and blue circles, respectively. The red curve shows the result of the least-squares fitting. In the conductance measurement, the gain of the current amplifier is switched from 10^9^ (in the STM measurements) to 10^5^ for measuring the large variation of the current in the tip approach and retraction processes. The currents in the almost flat region around label A in (b) are too small to be measured with this gain so that the conductance around A is nominally different from the value (2.6 × 10^−6^ *G*_0_) taken for *V*_S_ = 100 mV and *I*_t_ = 20 pA. (c) Histogram of *Z*_gap_max_. *Z*_gap_max_ is the maximum distance the tip travels before the SiNR nanojunction is broken after contacting the tip to the SiNR. (d) STM images before and after the conductance measurement. The scale bars correspond to 2 nm. (e) Two types of d*I*/d*V* spectra of the SiNR nanojunction. The spectra are measured with a modulation voltage of 4 mV at 312.6 Hz.

To reveal the intrinsic electronic features of SiNRs, we conducted transport measurements for individual SiNRs by lifting up each SiNR with the STM tip and fabricating a nanojunction consisting of an SiNR, the STM tip and the Ag substrate. This method reduces the SiNR–Ag interaction and enables us to reveal the intrinsic features of SiNRs. The measurements were performed by a scheme summarized in Figure 3a. At first, the STM tip is fixed over one end of the SiNR while the STM feedback loop is turned off. Then we approach the tip to the target SiNR while measuring the conductance *G* at the sample voltage of 100 mV as a function of tip vertical position (*Z*). We set the position where the tip is fixed initially as *Z* = 0. Once the tip touches the target, we retract the tip to lift up the SiNR and measure the d*I*/d*V* spectrum at certain tip position.

Figure 3b is an example of the conductance traces where the value of *G* is plotted as a function of *Z*. From the initial set point A to the point B, *G* increases as *Z* increases in the approaching procedure. The variation of *G* in this region is well fitted with an exponential function, as shown by the red curve, which is the calculated result of least-squares fitting. This indicates that the current flows through a vacuum gap. When the tip moves 0.54 nm, *G* suddenly increases from B (*G* = 0.2*G*_0_) to C (*G* = 1.4*G*_0_) where *G*_0_ is the conductance quantum (7.75 × 10^−5^ S). This discontinuous increase indicates that the tip touches the SiNR. Subsequently, we lift up the SiNR by retracting the STM to fabricate a nanojunction. When we lift up the SiNR further, the nanojunction is broken at D (*Z* = −0.4 nm) and *G* returns to the initial value of ≈10^−4^ *G*_0_ at E. In the retracting procedure from C to D, the nanojunction is preserved until the tip is retracted about 1 nm from the contact position. The conductance trace in this regime is different from that in the approach procedure. The conductance value remains higher than 0.1*G*_0_ until the junction is broken, indicating the conductance of the SiNR is almost comparable to a metallic nanowire. The resistance of a silicene field effect transistor (FET) is estimated to be about 40 kΩ from the drain current measured as a function of the drain voltage [24]. The sheet resistance of multilayer silicene sheets is measured to be 6.5 kΩ/□ [37]. These results also indicate that the silicene sheet is conductive, and the present results reasonably agree with the previous results. Figure 3c shows a histogram of the maximum gap distance, *Z*_gap_max_, which corresponds to the traveling distance of the tip from C to D. The histogram indicates how long the nanojunction can be fabricated. The nanojunctions are usually broken at small values of *Z*_gap_max_ and SiNRs can rarely be lifted up to 1.0 nm.

It is of interest to compare the properties of SiNRs with graphene nanoribbons (GNRs). The transport properties of armchair GNRs (AGNRs) grown on Au(111) have been investigated recently by using STM [38]. Similar to the present study, the conductance of individual AGNRs has been measured by lifting up each AGNR with an STM tip. The measured conductance values are the order of 10^−3^ *G*_0_, reflecting the semiconducting nature with a large energy gap. Comparing these results with those obtained in the present study, one can see that the SiNR is much more conductive, indicating that SiNRs would be a suitable material for a conducting wire used in nanostructured electronic devices. In contrast, the SiNR is not mechanically strong and the SiNR junction is more easily broken. The AGNR was able to be lifted up more than 3 nm. This may come from the stronger interfacial coupling between the SiNR and Ag(110) as well as the weaker bond strength of Si–Si bonding in SiNR than that of C–C bonding in AGNR. We also tried to lift up the narrower ribbons, but were not yet successful.

The d*I/*d*V* spectra of the SiNR nanojunction shows an interesting feature. Namely, we have found that a peak appears at the Fermi level as shown in the lower panel of Figure 3e. Note that the peak does not always appear in the spectra (as shown in the upper panel of Figure 3e), even though the SiNR is lifted up from the Ag(110) substrate. We measured more than 600 spectra for 250 SiNR nanojunctions. Whereas most spectra did not exhibit the remarkable structure as shown in the upper panel of Figure 3e, 31 spectra did show a clear peak structure at the Fermi level. As a reference, we measured the spectra of the nanojunctions which are fabricated by contacting the STM tip directly to the bare Ag(110) regions and lifting up the tip. We did not observe a peak structure for this type of nanojunction. As shown in Figure 3d, the structures are not drastically changed before and after the measurement except for a bright spot which arises from a small cluster dropped from the tip apex. Thus, we have concluded that the peak structure originates from the intrinsic properties of the SiNR.

Now let us examine the origin of the peak structure observed for the SiNR nanojunctions. Since the d*I/*d*V* spectrum essentially reflects the electronic density of states (DOS), we interpret the peak by comparing the d*I/*d*V* spectrum with the DOS spectra calculated for freestanding SiNR. The DFT studies have demonstrated that the geometric and electronic structures of SiNR strongly depend on the termination of dangling bonds at the edge Si atoms [29–30,33]. The electronic states localized at the edges appear near the Fermi level for the mono-hydrogenated SiNR in which the edge Si atoms are passivated with H atoms. In the case that the edge Si atoms are not terminated with H atoms, the honeycomb structure is unstable and vulnerable to the structural reconstruction at the edges. The DOS spectrum depends on the reconstructed structure. In the DFT study of Cahangirov et al. [29], the edge undergoes a reconstruction in which two deformed 6-membered rings are alternatively arranged along the edge. As a result, the flat band dispersion arising from the edge states disappears and instead a more dispersive band crosses the Femi level. In contrast, the DFT study of Ding and Wang [33] shows that a peak appears at the Fermi level in the DOS spectrum for a reconstructed SiNR in which the combination of six- and five-membered rings constitutes the edge. Assuming that the latter type of edge reconstruction takes place for the SiNR lifted by the STM tip, the peak structure can be rationalized by the DFT results of Ding and Wang. Finally, we briefly discuss the possibility of the pentagonal chain models proposed very recently for SiNRs on Ag(110) [27–28]. Cerdá et al. [27] calculated the energy band structures of various pentagonal chains; some of the pentagonal chains host electronic structure around the Fermi level, which may explain the peak structure observed in our conductance measurements. In the DFT calculation of Ding and Wang [33], pentagonal rings appear inside the honeycomb ribbon and the peak structure may reflect the pentagonal structure. However, further investigations are required to conclude the atomic structure and transport properties of SiNRs.

## Conclusion

We investigated the geometric and electronic structure of SiNRs grown on Ag(110) using STM and STM junction measurements. We found that the d*I*/d*V* spectra of SiNRs on Ag(110) and the bare Ag(110) regions are essentially identical, indicating strong interfacial coupling between the SiNR and the Ag(110) substrate, and that SiNR is a good conductor with conductance of 0.1*G*_0_–1*G*_0_. In addition, we have found a peak structure at the Fermi level for the SiNR nanojunctions, which is relevant to the edge of the SiNR.

## Experimental

All experiments were carried out in an ultra-high vacuum (UHV) chamber equipped with a low temperature STM (*P* < 10^−10^ Torr, *T* = 6 K). A Ag(110) single crystal surface was cleaned by repeated Ar ion sputtering and annealing at around 800 K. The STM tip was made of an electrochemically etched W wire and postannealed in the UHV chamber. The SiNRs were synthesized on Ag(110) by depositing Si atoms from the electrically heated Si wafer. The Ag(110) substrate was heated at 500 K during the Si deposition. The deposition rate was 0.03 ML/min, where 1 ML ≈ 1.5 × 10^15^ Si atoms/cm^2^. The differential conductance spectra (d*I*/d*V*) were measured by a lock-in technique with the modulation voltage of 0.4–8.0 mV at 300–500 Hz added to the sample voltage. The conductance measurements were carried out by lifting individual SiNRs with an STM tip and fabricating a nanojunction in which the SiNR bridges the STM tip and the substrate. The conductance measurements were made at 6 K. The typical tip approach/retraction speed was set at 0.06 nm/sec.

## References

[1] Nitzan A, Ratner M A (2003). Science.

[2] Joachim C, Ratner M A (2005). Proc Natl Acad Sci U S A.

[3] Tao N J (2006). Nat Nanotechnol.

[4] Hiraoka R, Arafune R, Tsukahara N, Kawai M, Takagi N (2014). Phys Rev B.

[5] Charlier J-C, Blase X, Roche S (2007). Rev Mod Phys.

[6] Rurali R (2010). Rev Mod Phys.

[7] Castro Neto A H, Guinea F, Peres N M R, Novoselov K S, Geim A K (2009). Rev Mod Phys.

[8] Kara A, Enriquez H, Seitsonen A P, Voon L C L Y, Vizzini S, Aufray B, Oughaddou H (2012). Surf Sci Rep.

[9] Takagi N, Lin C-L, Kawahara K, Minamitani E, Tsukahara N, Kawai M, Arafune R (2015). Prog Surf Sci.

[10] Oughaddou H, Enriquez H, Tchalala M R, Yildirim H, Mayne A J, Bendounane A, Dujardin G, Ali M A, Kara A (2015). Prog Surf Sci.

[11] Houssa M, Dimoulas A, Molle A (2015). J Phys: Condens Matter.

[12] Spencer M J S, Morishita T (2016). Silicene–Structure, Properties and Applications.

[13] Cahangirov S, Sahin H, Le Lay G (2017). Introduction to the Physics of Silicene and Other 2D Materials.

[14] Liu C-C, Feng W, Yao Y (2011). Phys Rev Lett.

[15] Ezawa M (2012). New J Phys.

[16] Ezawa M (2012). Phys Rev Lett.

[17] Lin C-L, Arafune R, Kawahara K, Tsukahara N, Minamitani E, Kim Y, Takagi N, Kawai M (2012). Appl Phys Express.

[18] Vogt P, De Padova P, Quaresima C, Avila J, Frantzeskakis E, Asensio M C, Resta A, Ealet B, Le Lay G (2012). Phys Rev Lett.

[19] Jamgotchian H, Colignon Y, Hamzaoui N, Ealet B, Hoarau J Y, Aufray B, Bibérian J P (2012). J Phys: Condens Matter.

[20] Feng B, Ding Z, Meng S, Yao Y, He X, Cheng P, Chen L, Wu K (2012). Nano Lett.

[21] Chiappe D, Grazianetti C, Tallarida G, Fanciulli M, Molle A (2012). Adv Mater.

[22] Arafune R, Lin C-L, Kawahara K, Kanno M, Tsukahara N, Minamitani E, Kim Y, Takagi N, Kawai M (2013). Surf Sci.

[23] Lin C-L, Arafune R, Kawahara K, Kanno M, Tsukahara N, Minamitani E, Kim Y, Kawai M, Takagi N (2013). Phys Rev Lett.

[24] Tao L, Cinquanta E, Chiappe D, Grazianetti C, Fanciulli M, Dubey M, Molle A, Akinwande D (2015). Nat Nanotechnol.

[25] Leandri C, Le Lay G, Aufray B, Girardeaux C, Avila J, Davila M E, Asensio M C, Ottaviani C, Cricenti A (2005). Surf Sci.

[26] Aufray B, Kara A, Vizzini S, Oughaddou H, Léandri C, Ealet B, Le Lay G (2010). Appl Phys Lett.

[27] Cerdá J I, Sławińska J, Le Lay G, Marele A C, Goméz-Rodríguez J M, Dávila M E (2016). Nat Commun.

[28] Prevot G, Hogan C, Leoni T, Bernard R, Moyen E, Masson L (2017). Phys Rev Lett.

[29] Cahangirov S, Topsakal M, Aktürk E, Şahin H, Ciraci S (2009). Phys Rev Lett.

[30] Ding Y, Ni J (2009). Appl Phys Lett.

[31] Kang J, Wu F, Li J (2012). Appl Phys Lett.

[32] Xu C, Luo G, Liu Q, Zheng J, Zhang Z, Nagase S, Gao Z, Lu J (2012). Nanoscale.

[33] Ding Y, Wang Y (2014). Appl Phys Lett.

[34] De Padova P, Quaresima C, Olivieri B, Perfetti P, Le Lay G (2011). Appl Phys Lett.

[35] De Padova P, Kubo O, Olivieri B, Quaresima C, Nakayama T, Aono M, Le Lay G (2012). Nano Lett.

[36] Feng B, Li H, Meng S, Chen L, Wu K (2016). Surf Sci.

[37] Vogt P, Capiod P, Berthe M, Resta A, De Padova P, Bruhn T, Le Lay G, Grandidier B (2014). Appl Phys Lett.

[38] Koch M, Ample F, Joachim C, Grill L (2012). Nat Nanotechnol.

